# How to Raise Birth-Rates after the War

**Published:** 1917-07-14

**Authors:** 


### HOW TO RAISE BIRTH-RATES AFTER THE WAR.

### Interesting Figures and Sober Facts.

This problem, which has exercised many minds of late, is touched on by Rohleder in the *Zeitschrift für Sexualwissenschaft* of April 1917. He begins by pointing out that not only is there the loss of men of reproductive ages to be reckoned with, but also economic impoverishment, which after the war will aggravate the existing strong tendency towards limitation of offspring. The pre-war causes of the decline in the birth-rate are then traced, a decline which in the last decade was to be observed in every European country save Portugal and Ireland. The economists Mombert and Brentano have shown that decline in birth-rate results primarily from increased prosperity. Statistics from Italy have shown that births were most numerous in the poorest districts, like Cosenza, and fewest in the richest, like Turin. Prosperity and high civilisation depress the birth-rate, poverty and primitive conditions raise it. Thus France had a birth-rate of 20.6 per thousand, England of 25, Norway of 26; but Serbia's was 39, Rumania's and Bulgaria's each 40, and Russia's 44. Births may thus be almost said to increase regularly as one passes across Europe from west to east, a fact the explanation of which is not to be found in climate, but in the civilisation level and the presence or absence of luxury. The second cause is the increasing industrialisation of most countries, whereby millions of women become wage-earners and are hampered as regards the function of wife- and mother-hood. Closely connected with this are urbanisation, the rush to town life, with consequent unhygienic conditions of dwellings; criminal abortion; venereal diseases; and lastly alcoholism—all things more or less attributable to the advance of civilisation. According to the author's experience, some of the causes commonly given for low birth-rate have no validity. Such are religious belief (Protestant Christianity as compared with Roman Catholicism or Hebraism) and decline in marriage-rate. The number of German marriages rose from 476,000 in 1900 to 513,000 in 1913. But in spite of this the births, although maintaining preponderance over the deaths, were 160,000 fewer. Nor will a European racial degeneration explain the facts, for infantile mortality has fallen as well as the births.

The author then inquires as to what measures may, in the light of the above facts, be considered as likely to be most effective. He recalls first some already suggested remedies—*e.g.*, a decided preference for married people in filling up State or municipal appointments, and as tenants, while bachelors should be made to give longer military service than married men. All such expedients, and a tax on bachelorhood, are rejected rather summarily as impracticable and ineffective. Money rewards for children are deemed worse than ineffective, because they would only act as incentives to the less desirable classes, whose rate of increase already outstrips that of higher grades of intelligence. "Social betterment" is a particularly unpromising remedy, because of the broad principle already mentioned, that improved pecuniary circumstances mean a low birth-rate. Rohleder's own idea is that the really fruitful measures may be divided into indirect, by which more children may be obtained, and direct, from which maintenance of the already existing population may be expected. The indirect means are the energetic combating of venereal disease, criminal abortion, and alcoholism; domestic hygiene; and abolition of the celibacy of the Catholic priesthood and of female school teachers. The direct ones are checking of emigration and of infantile mortality. Thus venereal disease is responsible for a number of sterile marriages, amounting to 12 per cent. of the whole; so that even on the one-child system nearly 200,000 children are lost yearly from this one cause. Leroy-Beaulieu has calculated that in France 100,000 criminal abortions are performed yearly. Municipal support of the anti-venereal campaign and the mother-protection and midwifery-aid movements must come into play as regards these two points. Alcoholism encourages prostitution and sexual precocity, while better dwellings and settlements on the land discourage them. Such land settlements should be at home. Abolition of religious celibacy would, in Germany, affect 41,000 men (priests and monks) and 76,000 women (nuns, etc.). As to emigration, this has always increased after wars. (The English reader will reflect in this connection upon the existence of conscription in the Mother Country, and its absence in some Colonies.) From Austro-Hungary emigration has always been very great. As to the campaign against infantile mortality, Dr. Drysdale and the neo-Malthusians are cited in support of the position that diminishing deaths is a much more practicable means of increasing the population of Europe than augmenting births. Germany has some leeway to make up, for *unser Gegner England* has an infantile mortality of 11.5, as against Germany's 17. Insurance by the State of mothers and infants, analogous to the existing national sickness insurance, is demanded, and it is claimed that a partial measure of this kind in 1915 reduced the infantile mortality, which in the first months of the war had begun to rise. So much for quantity; and to secure good quality of offspring incurable invalids and the like are to be enjoined to practise neo-Malthusian measures in their marriages, if they marry. Compulsory sterilisation of "degenerates," as to which laws have been passed in some North American States, is not recommended. Medical certificates of health for those contemplating marriage are, however, advised. The subject of this paper concerns, unfortunately, nearly every people in Europe. Of the breadth of the view here taken there is no doubt, and of course several of its recommendations are mooted in this country, and are perhaps begun. But when pecuniary incentives to child-bearing are given such short shrift, one thinks of the sensible-sounding words that someone—was it the expansive Mr. Wells?—said: "If the State wants children, it must pay for them."

